# Induction of Tyrosine Phosphorylation of UV-Activated EGFR by the Beta-Human Papillomavirus Type 8 E6 Leads to Papillomatosis

**DOI:** 10.3389/fmicb.2017.02197

**Published:** 2017-11-10

**Authors:** Stefanie Taute, Herbert J. Pfister, Gertrud Steger

**Affiliations:** Institute of Virology, University of Cologne, Cologne, Germany

**Keywords:** beta-HPV8, E6 oncoprotein, cutaneous squamous cell carcinomas, UV-light, papillomatosis, EGFR signaling and trafficking

## Abstract

Epidemiological evidence is accumulating that beta-human papillomaviruses (HPV) synergize with UV-light in the development of precancerous actinic keratosis, and cutaneous squamous cell carcinomas (cSCC), one of the most common cancers in the Caucasian population. We previously demonstrated the tumorigenic activity of beta-HPV type 8 (HPV8) in the skin of transgenic mice and its cooperation with UV-light. Analysis of underlying mechanisms now showed that in keratinocytes expressing the HPV8E6 protein a transient increase of tyrosine phosphorylated epidermal growth factor receptor (EGFR) in response to UV-irradiation occurred, while EGFR tyrosine phosphorylation, i.e., receptor tyrosine kinase (RTK)-activity was hardly affected in empty vector control cells. FACS and immunofluorescences revealed that the EGFR was internalized into early endosomes in response to UV-exposure in both, HPV8E6 positive and in control cells, yet with a higher rate in the presence of HPV8E6. Moreover, only in HPV8E6 expressing keratinocytes the EGFR was further sorted into CD63+ intraluminal vesicles, indicative for trafficking to late endosomes. The latter requires the ubiquitination of the EGFR, and in correlation, we could show that only in HPV8E6 positive keratinocytes the EGFR was ubiquitinated upon UV-exposure. HPV8E6 and tyrosine phosphorylated EGFR directly interacted which was enhanced by UV-irradiation. The treatment of K14-HPV8E6 transgenic mice with Canertinib, an inhibitor of the RTK-activity of the EGFR, suppressed skin papilloma growth in response to UV-irradiation. This confirms the crucial role of the RTK-activity of the EGFR in HPV8E6 and UV-mediated papillomatosis in transgenic mice. Taken together, our results demonstrate that HPV8E6 alters the signaling of the UV-activated EGFR and this is a critical step in papilloma formation in response to UV-light in transgenic mice. Our results provide a molecular basis how a beta-HPV type may support early steps of skin tumor formation in cooperation with UV-light.

## Introduction

The cutaneous squamous cell carcinoma (cSCC) is one of the most prevalent skin tumors in the Caucasian population and particularly frequent after organ transplantation. Since cSCC is a cancer of the elderly population with a mean age of 75 years it is estimated that its incidence will augment in the future due to the increased life expectation (Hillen et al., 2014). The precancerous actinic (solar) keratosis (AK) is an intraepidermal squamous neoplasm of sun-damaged skin. The transition to invasive cSCC is reported in 5–10% and with immunosuppression in 30% of patients. On the other side, 80% of the cSCC have AK with a variable grade of dysplasia in their periphery. Immunosuppressed organ transplant recipients (OTR) have a 100-fold increased risk to develop an invasive cSCC and 250-fold increased risk for AK (Lomas et al., 2012). Most AK and cSCC appear on sun exposed skin areas, which clearly points to the etiologic role of UV-light as initiator and tumor promoter of AK and cSCC. This relies on DNA damage including inactivating mutations of p53, and on effects of UV-light on signal transduction pathways that regulate cell proliferation and survival such as the signaling by the epidermal growth factor receptor (EGFR).

Evidence is accumulating that human papillomaviruses (HPV) belonging to the genus beta (β-HPV) synergize with UV-light in the development of cSCC. This was initially recognized in patients suffering from the rare disease Epidermodysplasia verruciformis (EV). CSCC in EV-patients harbor multiple, extrachromosomal genome copies of specific β-HPV types, especially HPV5 or HPV8 (reviewed in Howley and Pfister, 2015). β-HPVs are wide spread in humans but cause no apparent lesions in the skin of the general population. These viruses replicate in the hair follicles and are regarded as part of the commensal skin flora. Iatrogenic immunosuppression in OTR allows a more active replication of the commensal β-HPV spectrum in the entire skin with the result of higher viral load (Weissenborn et al., 2012). A high β-HPV DNA load in plucked eyebrow hairs was shown to imply a significant risk for the development of cSCC (Neale et al., 2013). The viral load of β-HPV in cSCC of the general population is lower compared to EV with usually less than 1 viral genome per cell (Weissenborn et al., 2005) and no viral transcripts have been detected (Arron et al., 2011). Though, the DNA loads of β-HPVs are higher in the precancerous AK (Weissenborn et al., 2005) and active viral replication and gene expression could be demonstrated (Borgogna et al., 2014). These findings are consistent with a role of β-HPV at early steps in non-melanoma skin cancer development by acting as a co-factor that enhances the carcinogenic potential of UV light.

The tumorigenic activity of β-HPV was demonstrated in transgenic mouse studies. We generated K14-HPV8CER transgenic mice that express the complete early region (CER) of HPV8 in the basal layer of their epidermis. Nearly all K14-HPV8CER transgenic mice spontaneously developed papillomas with varying degrees of epidermal dysplasia within 1 year. CSCC appeared in 6% of the animals (Schaper et al., 2005). After a single UV-irradiation or mechanical wounding, benign skin tumors grew within 3 weeks in all transgenic animals, which was consistently preceded by an enhanced expression of the viral oncogenes (Marcuzzi et al., 2009; Hufbauer et al., 2010). K14-HPV8E6 transgenic animals expressing only the E6 oncoprotein had the same phenotype as the K14-HPV8CER mice, indicating that HPV8E6 is the major oncogene in the murine skin (Marcuzzi et al., 2009). Transgene expression turned out to be crucial for tumor development since siRNA-mediated suppression of HPV8E6 reduced the papilloma incidence in these mice (Hufbauer et al., 2010). Thus, in this mouse model the HPV8 early genes cooperate with UV-light in the induction of skin tumors and the HPV8E6 oncogene is sufficient for this.

UV-irradiation transiently activates the EGFR which leads to increased proliferation, suppression of cell death and hyperplasia (El-Abaseri et al., 2006). Studies on mouse models deciphered the importance of EGFR signaling and downstream targets in the UV-induced skin tumorigenesis (Wan et al., 2001; El-Abaseri et al., 2005) and normal skin homeostasis (reviewed in Schneider et al., 2008). Ligand-, such as EGF, dependent activation initiates EGFR dimerization, which activates the receptor tyrosine kinase (RTK) resulting in the auto-phosphorylation of intracellular localized tyrosine residues of the EGFR. These phospho-tyrosines serve as binding motifs for a number of effectors which initiate the activation of downstream signaling pathways including the phosphatidylinositol 3 kinase (PI3K)/AKT, the Ras/mitogen-activated protein kinase (MAPK) pathways and Rac1 (Zhu et al., 2015) to promote cell survival and proliferation (reviewed in Lemmon and Schlessinger, 2010). Full activation of the EGFR as well as termination of the signaling depends on receptor endocytosis and intracellular trafficking. EGF-activated EGFR is rapidly internalized into early endosomes. Further downstream processing to late endosomes/lysosomes depends on the ubiquitination of the EGFR, which is initiated by binding of the E3 ubiquitin ligase Cbl to phospho-tyrosine residues of the EGFR. Ubiquitination is a prerequisite for the recognition by the endosomal sorting complex required for transport (ESCRT) that drives EGFR sorting from early endosomes via intraluminal vesicles (ILVs) to late endosomes and lysosomes leading to the proteasomal degradation. Dysregulation of this sorting process contributes to oncogenesis (reviewed in Tomas et al., 2014). Many intrinsic and iatrogenic cellular stresses such as UV-light, the cytokine TNFα or the cancer therapeutic cisplatin activate the EGFR in a tyrosine-kinase-independent way. UV-irradiation triggers a rapid clathrin-mediated internalization of the EGFR, which is ligand and RTK-activity independent and results in the accumulation of the UV-activated EGFR within the endosomal compartment without degradation (Zwang and Yarden, 2006).

Here, we addressed the effects of HPV8E6 on the UV-activated EGFR signaling and the implications for HPV8E6 mediated skin tumor formation in transgenic mice.

## Materials and Methods

### Mice

The K14-HPV8E6 mice were in the FVB/N background and express the HPV8E6 oncogene under control of the keratin 14 promoter (Marcuzzi et al., 2009). All mice were irradiated with UV as described previously at a dose of 1 J/cm^-2^ for UVB and 10 J/cm^-2^ for UVA (Marcuzzi et al., 2009). UV-radiation was generated by a UV device (UV 801; Waldmann). Prior to UV-exposure, mice were anesthetized and their back area was shaved with an electric shaver. A 2 cm^2^ area was irradiated while the rest of the skin was covered with a UV-impermeable sheet. The Canertinib group was administered once 20 mg/kg Canertinib (CI-1033, Selleckchem, S1019) solved in 150 μl PBS by gavage and the control group obtained 150 μl PBS 4 h prior UV-irradiation. After UV, the Canertinib group was treated with 10 mg/kg Canertinib solved in 150 μl PBS and the control group with 150 μl PBS daily for 24 days, respectively.

### Cell Culture

Normal human epithelial keratinocytes (NHEK) were obtained from PromoCell and were cultivated in KGM2 supplemented with growth factors and penicillin and streptomycin (S/P). HaCaT (provided by Professor N. Fusenig, German Cancer Research Center, Heidelberg, Germany) and C33A cells (from ATCC) were grown in DMEM. The immortalized keratinocyte cell line RTS3b [obtained from I. Leigh (Purdie et al., 1993)] and N/TERT (obtained from the ATCC) were cultivated in E-Medium. DMEM and E-medium were supplemented with 10% FCS and S/P. Transduction with pLXSN or pLXSN-HPV8E6 and selection by G418 was performed as previously protocol (Leverrier et al., 2007). RTS3b cells were transiently transfected with the X-treme gene reagent from Roche and C33A cells by the CaCl_2_ precipitate method. UV irradiation was performed at a dosage of 30 or 40 mJ/cm^2^ with UVB light using a UVP CL-1000 ultra-violet cross-linker with F8T5 bulbs giving a spectral peak at 320 nm. Cells were grown in media containing Canertinib, (4 or 7 μM), AG478 (10 μM) (Selleckchem) or vehicle for 2 h before UV-irradiation.

### Plasmids

The expression vectors pLXSN, pLXSN-HPV8E6, and pXJ41-FLAG-HPV8E6 have been described previously (Müller-Schiffmann et al., 2006; Leverrier et al., 2007). The expression vector for EGFR-GFP (Addgene Plasmid 32751) was a gift from Alexander Sorkin (Carter and Sorkin, 1998).

### Antibodies

The antibodies against the EGFR (D38B1) uncoupled or coupled to sepharose beads or to Alexa Flour (AF) 488, the phospho-EGFR-Y1068 (D7A5) and -Y1045 (#2237), -T669 (#8808) and the K48-polyubiquitin (12805), as well as the rabbit IgG isotype control (DA1E) were from Cell Signaling. The anti-EEA1 (BD Clone 14) and CD63 (BD clone H5C6) were purchased from BD Biosciences and M2-FLAG-affinity Gel and antibodies from Sigma and the GFP-tag antibody from Thermo-Scientific (A-11122). The guinea pig polyclonal antibody against HPV8E6 was kindly provided by Janet L. Brandsma and is described in Hufbauer et al. (2010).

### Western Blots and Co-IP

Cells were washed once in ice cold PBS and lyzed in low salt lysis buffer (20% glycerol, 50 mM Tris pH 7.9, 150 mM NaCl, 0.1% Nonidet-P40, 1 mM DTT) supplemented with protease inhibitors PMSF, aprotinin, leupeptin, and pepstatin and the phosphatase inhibitors NaF and Na-orthovanadate on ice for 15 min followed by sonification. IPs, blotting, and WB were done according to standard procedures. Blots were either developed with the use of X-ray films and scanned for digitalization or with the ChemiDoc XRS System (Bio-Rad, Dreieich, Germany), further processed with Adobe Photoshop CS6 (Adobe Systems Inc., Dublin, Ireland). Quantification was done with ImageJ software.

### Immunostainings and FACS-Analysis

Skin lesions were excised from transgenic mice, fixed in 4% paraformaldehyde and processed for paraffin embedding. Four micrometers thick sections were mounted and processed. IHC staining was performed with the Vectastain Universal Elite ABC Kit (Vector Laboratories, Peterborough, United Kingdom). Paraffin sections were stained for hematoxylin/eosin (H/E) and Giemsa following standard protocols. Sections were analyzed with the Leica DM4000B light microscope equipped with a KY-F75U digital camera (JVC) and Diskus 4.50 Software. For IFTs, cells were grown on coverslips, fixed in 4% paraformaldehyde for 15 min, washed with PBS three times and blocked for 60 min in PBS/10% horse serum/0.3% Triton-X-100, followed by incubation with the antibodies against EEA1 or CD63 (both diluted 1:200 in PBS, 5% horse serum, 0.15% Triton-X-100) for 2 h at rt. After three PBS washes for 5 min, the anti-mouse-IgG-Rhodamine (Santa Cruz), (1:100) and the anti-EGFR-AF488 (Cell Signaling, 1:150) antibodies were mixed and incubated for 2 h, followed by three PBS wash steps with DAPI being added to the last PBS wash. Coverslips were mounted on object plates and analyzed by a DMI6000B microscope equipped with a fluorescence Leica DFC365FX camera. The overlays were processed by the Leica LASX software. Pictures were further adapted with Adobe Photoshop CS6 (Adobe Systems Inc., Dublin, Ireland). Cells for flow cytometry were trypsinized, washed in PBS and fixed in 4% paraformaldehyde for 10 min at 37°C followed by 1 min on ice. After two washing steps with PBS/0.5% BSA, the cells were incubated with the anti-EGFR antibody (1:100) or the rabbit isotype control, both coupled to AF488 for 1 h at rt. After two times washing, the cells were resuspended in PBS and analyzed by FACS in a MACS Quant analyzer (Miltenyi, Germany) and the FlowJo software.

### RNA Processing

Skin samples were stored at -20°C in RNALater until RNA was isolated with the RNeasy kit (Qiagen, Hilden, Germany). One microgram of RNA was reverse transcribed using the GoTaq-RT-PCR System (Promega). Quantitative PCR were performed with the Go-Taq qPCR-Sybr-Green System (Promega) and a LightCycler480 (Roche).

### Ethics Statement

The generation of the transgenic mice and the UV irradiation protocols were approved by the governmental animal care office North-Rhine-Westphalia (protocol no. 84-02.04.2014.A087) and were in accordance with the German Animal Welfare Act as well as the German Regulation for the protection of animals used for experimental purposes.

## Results

### The Expression of HPV8E6 Leads to Increased Tyrosine Phosphorylation of the EGFR in Response to UV-Light in Keratinocytes

**FIGURE 1:**
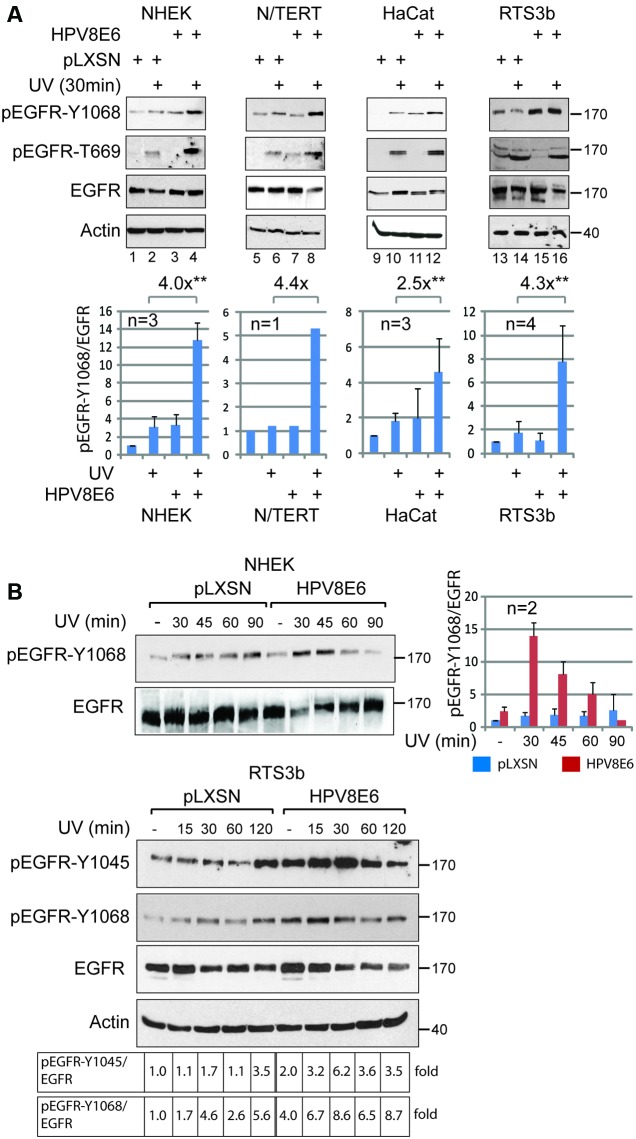
HPV8E6 increases the rate of tyrosine-phosphorylation of the EGFR after UV-irradiation. **(A)** Normal human epithelial keratinocytes (NHEK), N/TERT, HaCat and RTS3b cells, harboring either pLXSN or pLXSN-HPV8E6, respectively, were left untreated or irradiated with UV light and further cultivated for 30 min. Protein extracts were used for a WB developed with antibodies as indicated in the figure. Shown is one representative blot for each cell line. The signals were quantitated and the average ratio of pEGFR-Y1068 versus total EGFR obtained with three to four independent experiments, as indicated, were given in the graph for NHEK, RTS3b, and HaCat cells. The fold changes of the pEGFR-Y1068 versus total EGFR in UV-irradiated cell due to the expression of HPV8E6 are given. The ratios with N/TERTS are from one experiment (^∗∗^*p* < 0.05). **(B)** PLXSN-HPV8E6 or empty vector harboring NHEK and RTS3b cells were UV-irradiated and harvested at the indicated time points later. The pEGFR-Y1068, (pEGFR-Y1045 in the case of RTS3b) and the total EGFR were detected by WB. Shown is one representative example out of two experiments using NHEK, which both were used for quantifications shown in the figure. The values of the quantification of WB with the RTS3 cells are given as well. The positions of the molecular weight markers are given (in kDa).

To decipher the effects of UV and HPV8E6 on the activation of the EGFR we used keratinocytes expressing the HPV8E6 protein under control of the rather weak long terminal repeat (LTR) promoter of pLXSN-retroviruses to match the low level of the E6 protein as observed during natural HPV infections (Wallace et al., 2014). The expression of HPV8E6 in the stably transduced immortalized keratinocyte lines HaCat, RTS3b, and N/TERT, and in primary NHEK was confirmed by qRT-PCR (data not shown). All types of keratinocytes were kept 24 h under low serum conditions (i.e., 0.2% FBS), followed by UV-irradiation (30 mJ/cm^2^) and a recovery period of 30 min prior to be harvested. The stimulation of the RTK-activity in the UV-treated cells was analyzed by detecting the phosphorylation of the EGFR at tyrosine at pos. 1068 (pEGFR-Y1068) by WB. All unirradiated keratinocytes, except HaCat cells, had low, but detectable level pEGFR-Y1068 (**Figure 1A**, lanes 1, 5, and 13). These were hardly affected by UV-irradiation. In HaCat cells, pEGFR-Y1068 was only detectable after UV-irradiation. Importantly, in all four tested keratinocytes the signals corresponding to pEGFR-Y1068 were stronger in the presence of HPV8E6 compared those of empty vector cells 30 min after UV-irradiation (**Figure 1A**, lanes 4, 8, 12, and 16). The quantifications of the ratio of total EGFR versus pEGFR-Y1068 revealed that in the presence of HPV8E6 UV-exposure enhanced the RTK-activity of the EGFR, which ranged from 2.5-fold (in HaCat cells) to more than 4-fold (in RTS3b, N/TERT and primary keratinocytes). This implies that HPV8E6 increases the RTK-activity of the EGFR in response to UV-irradiation. We consistently observed a slight shift in the migration of the EGFR after UV-exposure (**Figure 1A**, compare lanes 1 and 2, 5 and 6, 13 and 14). This may be attributed to the UV-dependent phosphorylations of S or T residues which included the threonine at pos. 669 (T669). These phosphorylations have been found to be required for the internalization of the UV-exposed EGFR (Zwang and Yarden, 2006). The UV-light induced phosphorylation of the EGFR at T669 was confirmed in all four types of keratinocytes we investigated here (**Figure 1A**).

To address the effect of HPV8E6 on the tyrosine phosphorylation of the EGFR in more detail, we performed a kinetic. In empty vector control NHEK the level of pEGFR-Y1068 was hardly affected by UV-light within 60 min following the treatment. We regularly observed an enhanced tyrosine phosphorylation 90 or 120 min after UV-exposure in empty vector cells, which may be triggered by UV-induced p38MAPK activity, as suggested previously (Tomas et al., 2015). In HPV8E6 expressing primary keratinocytes, UV-irradiation led to a marked increase of the level of pEGFR-Y1068 30 min and 45 min after UV-treatment. Then, the amounts declined and finally returned to background level (**Figure 1B**, upper part). A similar kinetic of UV- and HPV8E6-mediated increase of the pEGFR-Y1068 was observed in RTS3b cell (**Figure 1B**, lower part). In addition, UV-irradiation transiently triggered the pEGFR-Y1045 detectable 30 min and 45 min later in HPV8E6 expressing cells. These results support the notion that the expression of HPV8E6 transiently increases the RTK-activity of the EGFR in response to UV-irradiation.

### HPV8E6 Alters the Intracellular Trafficking of the UV-Activated EGFR

**FIGURE 2:**
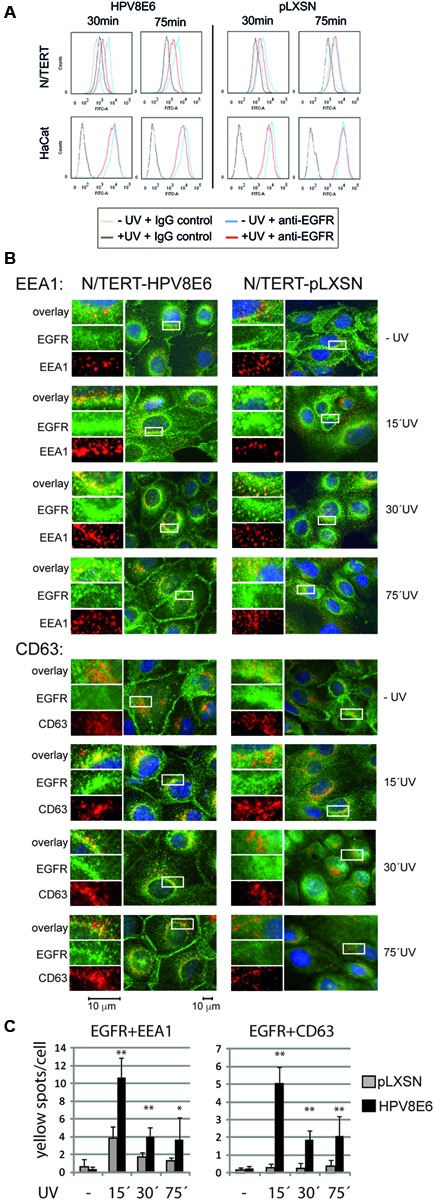
HPV8E6 alters the intracellular trafficking of the EGFR after UV-irradiation. **(A)** FACS analysis of fixed and non-permeabilized pLXSN-empty vector or HPV8E6-NTERT and HaCat cells. Cells were left untreated or irradiated with a dose of UV-light of 40 mJ/cm^2^ and harvested 30 or 75 min after UV-exposure. The cells were stained with the anti-EGFR-AF488 or the isotype control IgG-AF488 coupled antibody. The graphs represent the overlays of the EGFR-positive non-irradiated cells (blue line) with those harvested 30 min or 75 min post UV (red line), respectively.*(The light and dark gray graphs represent the cells stained with the isotype control. **(B)** Immunofluorescence analysis. N/TERT harboring the pLXSN or pLXSN-HPV8E6 were fixed and permeabilized after UV-exposure, as indicated. Immunofluorescence was performed with anti-EGFR-AF488 (green) and with the anti-EEA1 (red, upper part) or the anti-CD63 (red, lower part) antibody. Nuclei were stained with DAPI. The photos represent representative images, respectively. A more detailed section in a higher magnification is provided including the single stains. The scale bars are given at the bottom (magnification 1:1000). **(C)** Quantification of the number of EGFR positive early endosomes (EGFR and EEA1 positive), and of the CD63 and EGFR positive ILVs (in each case indicated by yellow spots). Bar graphs represent the fold changes of the number of yellow spots per cell, indicative for a co-localization of EGFR and EEA1 or CD63 15, 30, and 75 min after UV-irradiation. The values were obtained by counting the yellow spots in a total of 70 to 130 cells, obtained from two independent experiments. Error bars indicate the standard deviations, the asterisks indicate the significance (^∗∗^*p* < 0.001, ^∗^*p* = 0.052)*

UV-light induces an RTK independent EGFR transactivation and internalization, which results in an arrest within the early endosome without the ubiquitination and proteasomal degradation of the UV-activated EGFR within the first hour post-UV (reviewed in Tan et al., 2016). In order to investigate whether the internalization and the downstream sorting of the UV-activated EGFR was modulated by the expression of HPV8E6 we initially monitored the cell surface expression of the UV-exposed EGFR by FACS analysis with non-permeabilized N/TERT and HaCat keratinocytes. In both cell types, the amounts of EGFR at the cell surface were reduced 30 min after UV-irradiation, independently whether HPV8E6 was expressed or not (see **Figure 2A**). After a 75 min recovery period, however, in control cells the EGFR surface level returned to those as in untreated cells, while in HPV8E6 positive N/TERT and HaCat the amount of the surface EGFR remained reduced (**Figure 2A**). This implies that the intracellular processing of the UV-activated, internalized EGFR is altered in the presence of HPV8E6.

We further addressed this by immunofluorescence staining of UV-irradiated cells. In unirradiated control and HPV8E6 expressing N/TERT the EGFR was diffusely distributed along the plasma membrane (**Figure 2B**). It is noticeable that in HPV8E6 expressing keratinocytes a specific EGFR staining was detectable particularly at the adhesion sites, which was not further addressed here. Following UV-irradiation a substantial portion of the EGFR was redistributed from the plasma membrane into early endosomes, indicated by a co-localization of the early endosomal associated protein 1 (EEA1) and the EGFR (**Figure 2B**). This was detectable as early as 15 min up to 75 min post-UV exposure in both, control and HPV8E6 expressing cells, respectively (**Figure 2B**). Quantifications by counting the number of yellow spots within the cytoplasm per cell documented, however, that in the presence of HPV8E6 more EGFR was associated with early endosomes which was most obvious early after UV-irradiation (**Figure 2C**), implying that HPV8E6 enhances the internalization of the UV-activated EGFR.

To address the processing of the EGFR downstream of the early endosome we performed co-staining with CD63, also known as Lamp3, a well-established component of intraluminal vesicles (ILVs) and of late endosomal and lysosomal membranes. As early endosomes mature they can accumulate ILVs through inward budding of the membrane. The number of ILVs increases during endosome maturation. These finally fuse with the late endosomes and lysosomes and are then exposed to lysosomal hydrolysis (Oksvold et al., 2001). Interestingly, in the HPV8E6 positive cells a significant co-localization of CD63 positive structures and the EGFR was observed, indicated by the yellow spots which were not detectable in empty vector cells (**Figure 2B**). This was most pronounced 15 min after UV-irradiation, mostly at perinuclear sites. This co-localization progressively decreased 30 min and 75 min post UV, as confirmed by the quantification of the number of yellow spots (**Figures 2B,C**). No significant co-localization was obvious in the empty vector cells. Thus, UV-activated EGFR is further processed to ILVs in UV-irradiated HPV8E6 expressing cells while in control cells the UV-activated EGFR stays within the early endosome, in agreement with the findings described in the literature (Oksvold et al., 2001; Tomas et al., 2015).

### The Association of HPV8E6 with the EGFR Is Enhanced by UV-Light and Requires RTK-Activity

**FIGURE 3:**
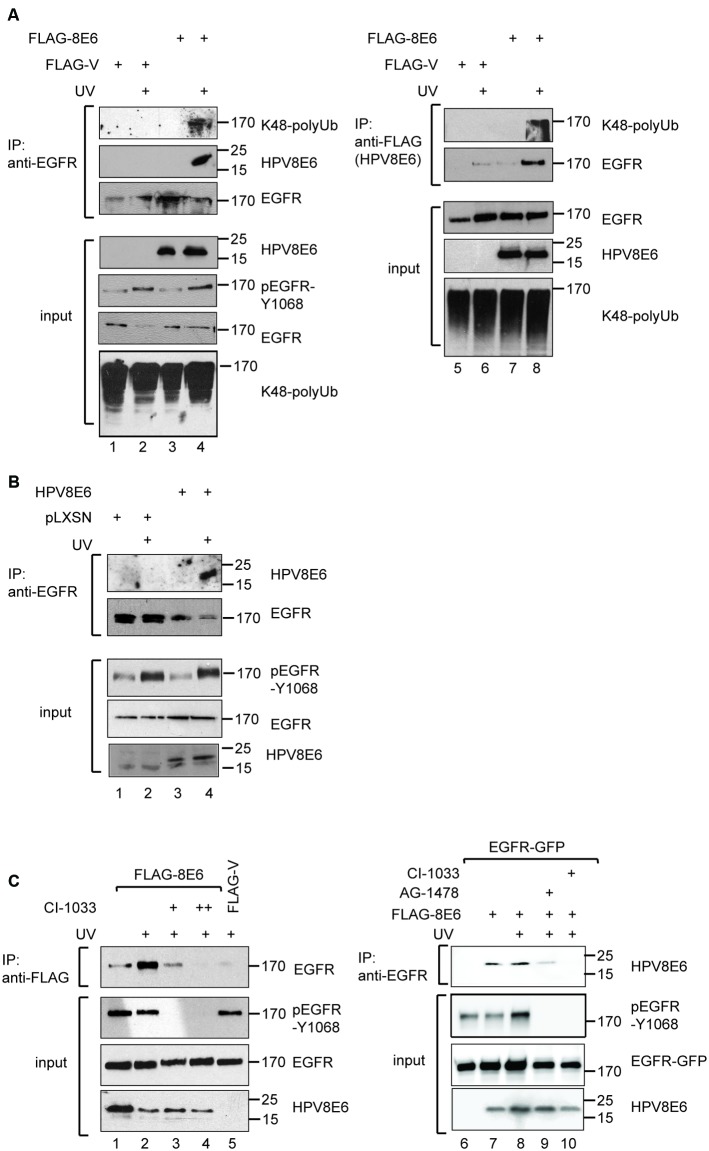
HPV8E6 directly binds to the ubiquitinated EGFR in an UV-dependent manner. **(A)** RTS3b cells were transiently transfected with a vector directing the expression of FLAG-tagged HPV8E6 under control of the CMV-promoter or the empty vector. Where indicated the cells have been irradiated with UV-light 30 min prior harvesting. 400 μg of extracts were incubated with the anti-EGFR-antibody (lanes 1–4) or with the FLAG-antibody (lanes 5–8), both coupled to sepharose, washed four times in 0.3 M KCl-buffer prior analysis by WB and 40 μg of the extracts were used in the input blots. **(B)** IP with anti-EGFR sepharose and 800 μg of extracts derived from the stable RTS3b-pLXSN and RTS3b-HPV8E6 lines, either left untreated or UV-irradiated 30 min prior harvesting. FLAG-HPV8E6 and EGFR present in the precipitate as well as in the input were detected by WB. **(C)** Extracts from UV-irradiated RTS3b cells that have been transiently transfected with the FLAG-HPV8E6 or the FLAG-vector were incubated either with DMSO (lanes 1, 2, 5), 3 μM (lane 3) or 7 μM (lane 4) Canertinib for 30 min. The IP was performed with the FLAG-antibody. All WB were developed with the indicated antibodies. In lanes 6–10, C33A cells were transiently transfected with expression vectors for EGFR-GFP and FLAG-HPV8E6, and UV-irradiated, where indicated, prior harvesting 30 min later. EGFR-GFP was precipitated from 400 μg of extract by the EGFR antibody and bound HPV8E6 was detected by the FLAG-antibody. The input blots were developed with the antibodies against pEGFR-Y1068, GFP, and FLAG. The positions of the molecular weight markers are provided (in kDa).

Ubiquitination of the EGFR is a prerequisite for ESCRT-mediated sorting into ILVs followed by proteasomal degradation of the tyrosine phosphorylated EGFR (Tomas et al., 2014). Since we observed that in the presence of HPV8E6 the UV-exposed EGFR is processed downstream of the early endosome into CD63 positive structures we analyzed whether the EGFR is ubiquitinated. We used UV-irradiated RTS3b cells, since these immortalized keratinocytes can be transiently transfected to high rate. After the transient transfection of a vector expressing FLAG-HPV8E6 under control of the strong CMV promoter the endogenous EGFR was precipitated. A subsequent WB with an antibody against K48-linked polyubiquitin-chains demonstrates that the EGFR underwent poly-ubiquitination in HPV8E6 positive cells after UV-exposure (**Figure 3A**, lane 4), in contrast to cells transfected with the empty vector. Beyond that, probing the blot with the antibody against HPV8E6 revealed that HPV8E6 specifically co-precipitated with the EGFR after UV-irradiation. This was confirmed by a co-immunoprecipitation of the EGFR with the FLAG-antibody, recognizing the FLAG-tagged HPV8E6 (**Figure 3A**, lanes 5–8). Again, the HPV8E6-associated EGFR was highly ubiquitinated. A specific interaction between the EGFR and HPV8E6 in response to UV-irradiation could also be demonstrated with extracts from the RTS3b-pLXSN-HPV8E6 cell line, which expresses much lower levels of HPV8E6 (**Figure 3B**). These results demonstrate that UV-irradiation enhances the binding of HPV8E6 to the endogenous EGFR and that the HPV8E6-associated EGFR is ubiquitinated. Since the ubiquitination of the EGFR requires its prior tyrosine phosphorylation, we analyzed whether the RTK-activity modulates the interaction with HPV8E6 and cultivated the transiently transfected cells in medium containing the irreversible RTK-pan-ErbB-inhibitor Canertinib, also known as CI-1033 (Smaill et al., 2000). As shown in **Figure 3C**, the binding of HPV8E6 to the EGFR was abolished in a dose dependent manner in the presence of Canertinib even when the cells have been exposed to UV-light. No tyrosine phosphorylated EGFR could be detected in the input confirming the efficiency of the RTK-inhibitor. The amount of total EGFR and HPV8E6 were hardly affected by Canertinib and by UV-irradiation, respectively (**Figure 3C**). We further confirmed these results with recombinant proteins. C33A cells were transiently transfected with expression vectors for GFP-tagged EGFR and FLAG-HPV8E6 and treated in addition to Canertinib also with the EGFR specific inhibitor tyrphostin AG-1478 followed by UV-irradiation. AG-1478 competitively binds to the ATP pocket of the EGFR to inhibit its RTK-activity (Gan et al., 2007). Here, FLAG-HPV8E6 co-precipitated with the GFP-tagged EGFR. This interaction was clearly reduced in the presence of AG-1478 and abolished by Canertinib. It has to be mentioned that the endogenous EGFR present in C33A cells could not be precipitated and detected under these conditions (data not shown). The residual binding may result from marginal Y-1068 phosphorylation of the overexpressed EGFR in the presence of AG-1478, which was still visible after long exposures (data not shown). Thus, HPV8E6 directly binds to the EGFR, which requires its Y-phosphorylation. From this we conclude that HPV8E6 enhances the RTK-activity of the UV-exposed EGFR, which promotes its interaction with HPV8E6 and leads to ubiquitination and to altered intracellular trafficking of the EGFR.

### Inhibition of the RTK-Activity of the EGFR Suppresses UV-Induced Skin Papillomatosis in K14-HPV8E6 Mice

**FIGURE 4:**
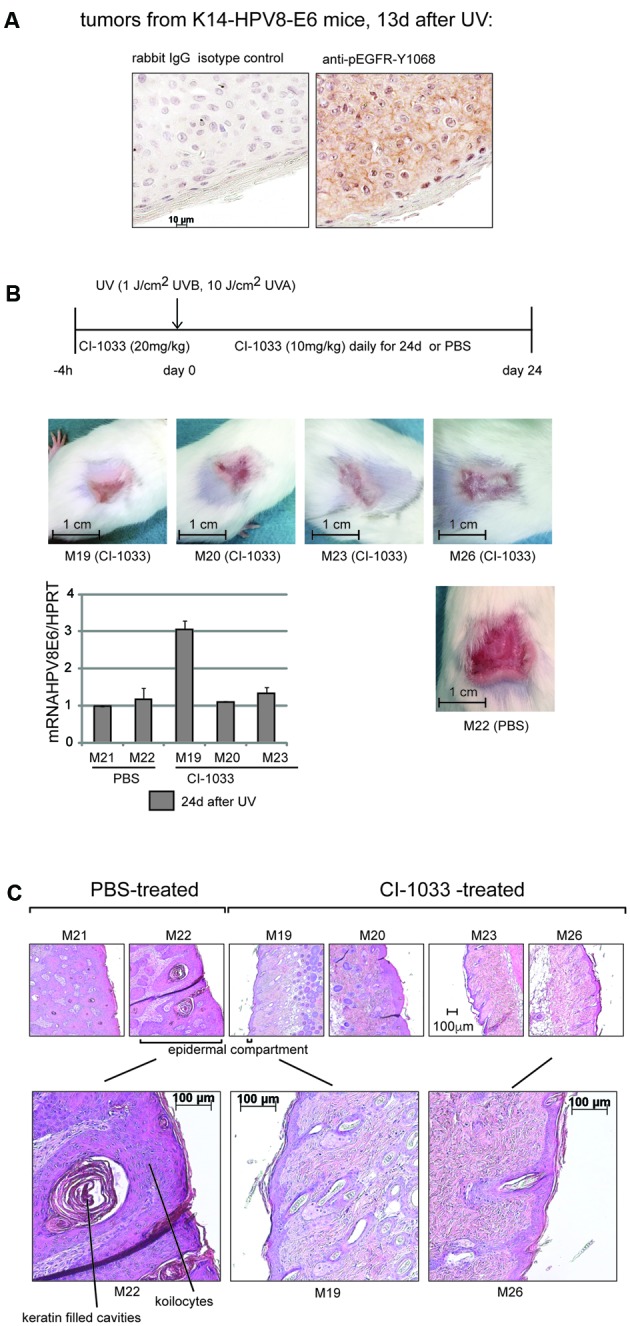
Inhibition of the RTK-activity of the EGFR by the pan-ErbB-inhibitor Canertinib (CI-1033) suppresses UV-induced papillomatosis in K14-HPV8E6 transgenic mice. **(A)** Immunohistochemical staining of paraffin embedded sections obtained from skin of K14-HPV8E6 transgenic mice 13 days after UV-exposure with the anti-pEGFR-Y1068 or rabbit IgG isotype control (magnification 1:400). **(B)** Transgenic mice obtained either Canertinib (CI-1033) (20 mg/kg weight) or the same volume of PBS 4 h prior UV-irradiation followed by applications of Canertinib once daily at a dose of 10 mg/kg weight or 150 μl PBS for 24 days, as given in the time line. The pictures represent examples of the lesions from four mice treated with CI-1033 (M19, 20, 23, and M26) and from one control mouse which obtained PBS (M22). The graph beneath shows the quantification of RT-PCR results with RNA isolated from two PBS and three Canertinib-treated animals to demonstrate the expression of HPV8E6. **(C)** Histology with H/E staining of skin section obtained from two PBS-control (M21 and M22) and four Canertinib (CI-1033)-treated animals (M19, M20, M23, and M26) 24 days after UV-irradiation.

We asked whether the activation of the RTK-activity of EGFR by HPV8E6 is relevant for the induction of papilloma growth in transgenic mice. Initially, we determined the level of pEGFR-Y1068 in hyperplastic skin lesions that appeared 13 days after UV-irradiation in K14-HPV8E6 transgenic mice by immunohistochemistry with the specific antibody. A faint specific staining of the epidermal cells associated with the plasma membranes and intracellular sites was readily visible indicating that the EGFR is in a tyrosine phosphorylated state (**Figure 4A**). To address its functional contribution to HPV8E6 and UV-mediated tumor formation, we treated K14-HPV8E6 transgenic mice with the RTK-inhibitor Canertinib. Seventeen K14-HPV8E6 transgenic mice obtained orally once 20 mg/kg body-weight Canertinib 4 h prior UV-irradiation and then once daily 10 mg/kg Canertinib for a period of 24 days while 12 UV-irradiated K14-HPV8E6 transgenic animals, representing the control group, were administered the solvent PBS at the same time points (see the time scale in **Figure 4B**). All K14-HPV8E6 transgenic mice as well as two wt mice (which were included as control) developed a sun burn 2–3 days after UV-irradiation according to our previously established protocol (Marcuzzi et al., 2009). While the sun burn healed after about 7 days in the wt mice, as described previously (Marcuzzi et al., 2009), papillomas started to grow in the transgenic mice demonstrating the effect of HPV8E6 which is expressed in the skin of these animals. After 24 days, all 12 animals in the control group had developed large, exalted papillomas covering the initially irradiated skin sections. In contrast, in all 17 animals treated with Canertinib the initially UV-irradiated skin-areal, which had developed a sun burn, had shrunk and none of the lesions was exalted (**Figure 4B**). The affected skin within the irradiated area appeared to be renewed and tender. RT-PCR with RNA isolated 24 days after UV-treatment from the lesions excluded that the expression of the HPV8E6 was diminished due to the application of Canertinib (**Figure 4B**). H/E-staining of lesions from two PBS treated animals revealed histology of papillomatosis, with multiple cavities filled with lamellated concentric keratin masses in the epidermal compartment. In the upper layers, intracytoplasmic keratohyalin granules were present and in some areas koilocytosis occurred, as observed previously (Schaper et al., 2005; Marcuzzi et al., 2009). The histology of the lesions from four animals of the Canertinib group illustrates that the epidermis from three Canertinib treated animals (M19, M23, and M26) was composed of two to three cell layers (**Figure 4C**). In the section of one treated mouse, the epidermal compartment was thickened but was still much thinner compared to the lesions of the control animals (M20, **Figure 4C**). These results demonstrate that the RTK-activity of the EGFR is required for UV-induced papilloma formation in K14-HPV8E6 transgenic mice.

## Discussion

We demonstrate here that HPV8E6 transiently increases the RTK-activity of the EGFR shortly after UV-exposure. We observe an increased tyrosine phosphorylation of the EGFR as a consequence of the expression of HPV8E6 in three different immortalized keratinocyte lines as well as in primary keratinocytes. This excludes cell-lineage dependent effects and points to a specific impact of HPV8E6 on the RTK-activity. We provide evidence that the effect of HPV8E6 has consequences on the downstream processing of the EGFR. The UV-activated EGFR is ubiquitinated only in HPV8E6 positive cells indicating that the HPV8E6-dependent increased tyrosine phosphorylation of the UV-exposed EGFR is sufficient to recruit the E3 ubiquitin-ligase Cbl and its associated Grb2. Indeed we observed a co-precipitation of Grb with the UV-stimulated EGFR in the presence of HPV8E6 (data not shown). Moreover, there was an enhanced internalization of the UV-activated EGFR into early endosomes in HPV8E6 cells compared to control cells and the presence of the EGFR in structures downstream of the early endosome even entirely depended on HPV8E6.

Tyrosine phosphorylated, ubiquitinated and endocytosed EGFR was shown to interact with Eps15 and AP-2, two endocytotic adaptor proteins to form clathrin coated endocytic vesicles (Zwang and Yarden, 2009). The complex is then targeted by the ESCRT and further sorted to CD63 positive ILVs beyond the early endosome, which fuse with lysosomes to complete the degradation of the EGFR and attenuate the signaling (Tomas et al., 2014). This supports the notion that UV-activated EGFR is prone to lysosomal degradation in HPV8E6 expressing cells, and indeed we occasionally observed reduced amounts of EGFR in the HPV8E6 cells up to 30 min after UV-irradiation where the highest level of tyrosine phosphorylation was present (see **Figure 1A**, lanes 7, 8, 15, 16 and **Figure 1B**). The distinct mode of endosomal EGFR trafficking induced by HPV8E6 was underlined by the lack of detectable ubiquitination and of co-localization with CD63 of the UV-activated EGFR in HPV8E6 negative cells. Thus, in agreement with previous reports the UV-light resulted in EGFR internalization with an arrest in the early endosome in HPV8E6 negative cells (Tomas et al., 2014; Peng et al., 2016).

The altered intracellular trafficking of the UV-activated EGFR induced by HPV8E6 may prolong the signaling and support cell survival. Activation of EGFR-signaling pathways not only occurs from the plasma-membrane. For instance, EGFR endocytosis to early endosomes was found to be required for the full stimulation of EGF-induced ERK1/2 and PI3K/AKT signaling (Vieira et al., 1996). Particularly, perinuclear endosomes have been shown to provide a spatial compartment for prolonged EGFR signaling (Huang et al., 2016). Our data showing a co-localization of the UV-activated EGFR and CD63 predominantly at perinuclear regions in HPV8E6 N/TERTs may thus reflect a prolonged signaling. A stimulation of the EGFR-mediated MAPK-Ras signaling pathway was suggested to occur from late endosomes and lysosomes as well (Oksvold et al., 2001; Jiang and Sorkin, 2002; Fehrenbacher et al., 2009; German et al., 2011). Moreover, continuous EGFR signaling from late endosomes was shown to contribute to sustained downstream AKT and STAT3 activation and the endosomal accumulation of the activated EGFR increased tumor cell survival (Huang et al., 2016).

In accordance with these observations, we report here that the functional interaction between HPV8E6 and the UV-activated EGFR which we have characterized here has a crucial role in the UV- and HPV8E6-induced skin tumor induction *in vivo* since the pharmacological inhibition of the RTK-activity of the EGFR suppressed papillomatosis in transgenic mice. The contribution of HPV8E6 is reflected by the facts that the UV-induced skin lesions, i.e., sun burn, healed in wt mice, while in HPV8E6 positive mice a massive tumor growth was induced, as observed previously (Marcuzzi et al., 2009). Since these mice have the same phenotype as the K14-HPV8CER mice, expressing all early HPV8 proteins, it is obvious that HPV8 E6 is the major oncogene in the mouse skin and can function on its own to stimulate papilloma growth in cooperation with UV-light. In addition, it is also necessary since its suppression reduced tumor formation in the HPV8CER mice (Hufbauer et al., 2010). Nevertheless, it has to be admitted that with this setting we used here potential interaction between the other early viral proteins E7, E2 and E1 with E6 or with the EGFR or the corresponding signaling pathways, which may occur during natural infections, have not been considered and cannot be excluded.

A previous study suggested that the short lived UV-induced activation of the EGFR is a powerful promoter of skin tumorigenesis of initiated keratinocytes (El-Abaseri et al., 2005). Similarly, the HPV8E6-mediated transient boost of the RTK-activity of the EGFR may support papilloma growth. It has to be mentioned that although our *in vivo* results obtained with transgenic mice imply that the functional stimulation of the EGFR-activity by HPV8E6 is essential for tumor induction, they do not prove it. The pan-ErbB inhibitor Canertinib has highest activity against the EGFR but also blocks the activity of ErbB2/Her2, which was shown to be expressed and to be tumorigenic in mouse skin as well (Kiguchi et al., 2000; Krahn et al., 2001). Thus, it cannot be excluded that a possible functional interaction of HPV8E6 with ErbB2 may contribute to the UV-induced papilloma formation.

What might be the molecular basis for the HPV8E6-mediated enhancement of the UV-activated EGFR? In agreement with published data (Zwang and Yarden, 2006), we found that UV-treatment induced S/T phosphorylations including that of the T in pos. 669, which were shown to be dependent on the UV-activated p38MAPK. These triggered a gradual and weak transient RTK-activation of the internalized EGFR which was detectable within the following 2–4 h (Tomas et al., 2015). However, others have reported weak tyrosine phosphorylations within minutes after UV-exposure (El-Abaseri et al., 2006; Singh et al., 2009). Since the binding of HPV8E6 to the EGFR depended on its tyrosine phosphorylation and was enhanced by UV-light, one might speculate that low level of tyrosine phosphorylations early after UV-exposure will initiate the recruitment of HPV8E6. This may somehow stabilize the phosphorylated EGFR form by a not yet characterized feedback mechanism. It has been reported that EGFR ubiquitination requires a certain threshold of receptor tyrosine phosphorylations, namely the simultaneous presence of two phosphotyrosines, of the pY1045 and either one of pY1068 or pY1086, on the same EGFR moiety. These mechanistically led to the cooperative recruitment of the E3 ligase Cbl in complex with Grb2, to the EGFR (Sigismund et al., 2013). Thus, HPV8E6 might promote to pass the required threshold of tyrosine phosphorylation allowing then EGFR-ubiquitination, with the consequence of altered endosomal trafficking and signaling of the UV-activated EGFR as discussed. Further studies are necessary to elucidate the mechanism how HPV8E6 stimulates the RTK-activity of the EGFR and whether the interaction will affect the activity of E6. Moreover, a functional interaction of HPV8E6 with other ErbB family members may participate, as discussed above, which has to be analyzed as well. The observation that the pharmacological inhibition of the RTK-activity suppressed papillomatosis mediated by HPV8E6 and UV-light was surprising in view of multiple, pleiotropic effects described for β-HPV E6 proteins. In common they have the capacity to inhibit MAML, which acts as co-factor for Notch. In this way, E6 may suppress keratinocyte differentiation via inhibiting Notch (Tan et al., 2012; Meyers et al., 2013) and thus further amplify the negative effect of EGFR signaling on Notch (Kolev et al., 2008). The E6 proteins of HPV8 and other β-HPV-types are able to inhibit apoptosis (Jackson and Storey, 2000; Jackson et al., 2000; Struijk et al., 2008) and interfere with the repair of UVB-induced DNA damage and repair (Wallace et al., 2012; Hufbauer et al., 2015) and thus favor the accumulation of mutations including UV-induced inactivating mutations of p53, which are thought to be a crucial step in the development of AKs and cSCC also in humans (Brash et al., 1991; Kemp et al., 1993; Jiang et al., 1999). These broader activities of HPV8E6 may support the progression of the benign papillomas, which depend on the RTK-activity of the EGFR as we have demonstrated here, to cSCC (Schaper et al., 2005; Marcuzzi et al., 2009).

Targeting the EGFR signaling seems to be a common theme for HPV. Previously, the E5 and the E6 oncoproteins of the high risk genital HPV16 belonging to the genus alpha were found to interfere with EGF stimulated EGFR signaling pathways and thus protect cells from apoptosis and induce migration, respectively (Zhang et al., 2005; Spangle and Munger, 2013). Here, we demonstrate for the first time, to our best knowledge, a functional interaction of an HPVE6 protein with the UV-activated EGFR. Remarkably, β-HPV types do not encode an E5 protein and E6 may have overtaken the role to modify signaling by UV-activated EGFR. This may reflect the association of β-HPV with the sun exposed cutaneous skin. It will be interesting to analyze whether other β-HPVE6 proteins are able to bind the Y-phosphorylated EGFR and to stimulate the UV-dependent activation of the EGFR as well and whether this is linked to E6 proteins of HPV types that are cancer associated.

Our data obtained with the use of transgenic mice and human keratinocytes provide a molecular basis how HPV8 may cooperate with UV-light in the induction of papillomas and also support a role of β-HPV in the development of proliferative skin lesions in humans, as implied by epidemiological studies. These findings also provide a molecular mechanism explaining the contribution of β-HPVs to the early steps in the cSCC development in human, particularly in immunosuppressed patients. In these patients, higher loads of β-HPV in the skin and the hair follicles due to their immune-suppression will result in elevated levels of E6, which then alter UV-induced EGFR signaling according to our data. Although this effect will be transient, reiterated UV irradiation may lead to sustained activation of the EGFR and downstream signaling pathways and thus trigger hyperproliferation of the epidermis and the development of AK, the precursor lesions of cSCC.

## Author Contributions

ST, HP, and GS planned and designed the experiments. ST conducted most of the experiments and analyzed the results. GS conducted and analyzed some of the experiments. GS wrote the manuscript. All the authors reviewed the manuscript.

## Conflict of Interest Statement

The authors declare that the research was conducted in the absence of any commercial or financial relationships that could be construed as a potential conflict of interest.
